# International Active Surveillance Study of Women Taking Oral Contraceptives (INAS-OC Study)

**DOI:** 10.1186/1471-2288-9-77

**Published:** 2009-11-18

**Authors:** Juergen C Dinger, Kristina Bardenheuer, Anita Assmann

**Affiliations:** 1 ZEG - Berlin Center for Epidemiology & Health Research, Invalidenstrasse 115, 10115 Berlin, Germany

## Abstract

**Background:**

A 24-day regimen of contraceptive doses of drospirenone and ethinylestradiol (DRSP/EE 24d) was recently launched. This regimen has properties which may be beneficial for certain user populations (e.g., women suffering from premenstrual dysphoric disorder or acne). However, it is unknown whether this extended regimen has an impact on the cardiovascular risk associated with the use of oral contraceptives (OCs). The **IN**ternational **A**ctive **S**urveillance study of women taking **O**ral **C**ontraceptives (INAS-OC) is designed to investigate the short- and long-term safety of the new regimen in a population which is representative for the typical user of oral contraceptives.

**Methods/Design:**

A large, prospective, controlled, non-interventional, long-term cohort study with active surveillance of the study participants has been chosen to ensure reliable and valid results. More than 2,000 gynecologists in the US and 5 European countries (Austria, Germany, Italy, Poland, and Sweden) will recruit more than 80,000 OC users. The two to five year follow-up of these women will result in at least 220,000 documented women-years.

The main clinical outcomes of interest for the follow-up are deep venous thrombosis, pulmonary embolism, acute myocardial infarction and cerebrovascular accidents. Secondary objectives are general safety, effectiveness and drug utilization pattern of DRSP/EE 24d, return to fertility after stop of OC use, as well as the baseline risk for users of individual OC formulations.

Because of the non-interference character of this study, potential participants (first-time users or switchers) are informed about the study only after the decision regarding prescription of a new OC. There are no specific medical inclusion or exclusion criteria. Study participation is voluntary and a written informed consent is required. After the baseline questionnaire, follow-up questionnaires will be mailed to the participants every 6 months for up to 5 years after baseline. Self-reported serious adverse events will be validated by contacting the relevant physician and by reviewing relevant source documents. At the end of the study an independent blinded adjudication of relevant clinical outcomes will be conducted.

Meanwhile, this study has received ethical approval from the Western Institutional Review Board (USA) and the Medical Association in Berlin (Germany).

**Discussion:**

The feasibility of the study is considered to be very high because of its similar design to the EURAS-OC study. All relevant methodological and logistical features of the study were successfully tested in the EURAS study.

The chosen design minimizes the impact of referral and misclassification bias, healthy user effect and loss to follow-up. Overall, it is expected that the study design is robust enough to interpret hazard ratios of 1.5 or higher.

## Background

Since their introduction in the mid-1960s, oral contraceptives have become a very popular method of birth control. Their safety has been improved over the years with the reduction in the doses of estrogen and progestogen. However, concerns about their safety have remained, peaking in the mid-1990s with the discussion on whether so-called "third generation" progestogens (desogestrel and gestoden) have a higher risk of cardiovascular side effects (especially venous thromboembolism - VTE) than older formulations. A number of studies have been published with conflicting results. Most of these studies have substantial methodological shortcomings and the discussion on the impact of bias and confounding on the results has not been resolved [1-17].

In the early 2000s the new progestogen drospirenone (DRSP) was introduced. Five studies investigated the VTE risk associated with the combination of 3 mg DRSP and 30 mcg ethinylestradiol (EE) given for 21 days per cycle (DRSP/EE 21d). Two large prospective cohort studies came to the conclusion that the combination of 3 mg DRSP and 30 mcg ethinylestradiol (EE) given for 21 days per cycle (DRSP/EE 21d) are as safe as OCs containing EE and levonorgestrel with regard to venous thromboembolism (essentially deep venous thrombosis and pulmonary embolism) and arterial thromboembolism (essentially acute myocardial infarction and cerebrovascular accidents) [12,13]. A case-control study in The Netherlands [14] and a large retrospective Danish cohort study using information from the Danish registries [15] found that DRSP might increase the risk for VTE compared to levonorgestrel-containing combined oral contraceptives. However, the results of the Dutch study were not statistically significant and the risk estimates of the Danish study were not adjusted for important confounders. In addition, the Danish study underestimated the VTE risk associated with the use of levonorgestrel-containing preparations because of differential misclassification of duration of use. The fifth study - a German case-control study [16] did not find an increased risk for DRSP/EE 21d, but the results have been published only as an abstract. A more detailed review of the four studies is published elsewhere [17]. All five studies investigated the 21-day regimen of DRSP. Overall, the existing evidence suggests that the VTE risk associated with the use of DRSP/EE 21d is similar to the VTE risk associated with OCs containing other progestogens.

This study investigates a 24-day regimen of a combination of 3 mg DRSP and 20 mcg EE (DRSP/EE 24d): 24 days of active pills are followed by 4 days of placebo pills. Based on the lower estrogen dose of DRSP/EE 24d, it can be assumed that a 21-day regimen of this pharmaceutical formulation would not be associated with a higher risk of venous thromboembolism (VTE) than DRSP/EE 21d.

Clinical studies indicate that a 24-day regimen of contraceptive doses of drospirenone and ethinylestradiol (DRSP/EE 24d) leads to stable hormone levels in the blood and a strong suppression of ovarian activity [18]. In addition, DRSP has antiandrogenic and antimineralocorticoid properties [19]. Compared to other oral contraceptives (OC) the new regimen may lead to better contraceptive effectiveness and better control of premenstrual symptoms.

It is unknown, however, whether the extended 24-day regimen has an impact on the cardiovascular risk associated with the use of oral contraceptives. Though the 24-day regimen is not expected to have a negative impact on the risk of VTE and arterial embolism (ATE) compared to a 21-day regimen, a study to assess this impact was deemed appropriate. It is conceivable that the reduction in so-called hormone swings leads to a lower incidence of venous thromboembolism. It is also conceivable, however, that the higher cumulative doses of progestin and estrogen lead to a higher risk. This non-interventional post-authorization safety study (PASS) is a phase IV commitment to the FDA. The study proposed in this protocol should provide data that are sufficiently robust to show that there is no increase in VTE risk for DRSP/EE 24d.

The EURAS study has demonstrated that a large, prospective, controlled, non-interventional, long-term cohort study is suitable for

1. Safety monitoring of an oral contraceptive

2. Reliable identification of relevant clinical outcomes and

3. Providing robust estimates of their incidence.

The INAS-OC Study has a similar study design but the procedures for recruitment, informed consent and follow-up were slightly modified to comply both with European and US regulations, and to ensure good recruitment rates and low loss to follow-up in a transatlantic environment.

The study should provide early information and regular updates on relevant clinical outcomes which will contribute to a continuous risk - benefit assessment during long-term follow up (3 to 5 years in the US, 2 to 4 years in Europe).

The US part of the study started already in August 2005, because of the early launch of DRSP/EE 24d in the US. The European part of the study started in fall 2008 with the market introduction of DRSP/EE 24d in Europe.

The primary objective of the study is to assess the risks of short and long-term use of DRSP/EE 24d and of established OCs in a study population that is representative for the actual users of the individual preparations. This includes an estimate of the absolute risk of rare serious adverse outcomes.

The main clinical outcomes of interest for the short and long-term follow-up are:

• Venous Thromboembolism (VTE; mainly Deep Venous Thrombosis (DVT) and Pulmonary Embolism (PE))

• Arterial Thromboembolism (ATE; mainly Acute Myocardial Infarction and cerebrovascular accidents (CVA))

Secondary objectives are:

• to investigate the general safety and effectiveness of DRSP/EE 24d under real-life conditions in typical user populations (e.g., obese women, adolescents)

• to investigate return to fertility in users who stop OC use because of a planned pregnancy

• to analyze the drug utilization pattern of DRSP/EE 24d and established OCs in a study population that is representative for typical use of the individual preparations under routine medical conditions in Europe and the United States. Interference of study-specific requirements and measures with the normal drug utilization pattern should be minimized by using a non-experimental study design

• to characterize the baseline risk of users of the individual formulations (lifetime history of co-morbidity, risk markers, co-medication, socio-demographic and lifestyle data)

## Methods/Design

The INAS-OC Study is a large, transatlantic, prospective, controlled, non-interventional, long-term cohort study that follows a series of cohorts. The cohorts consist of new users (first-ever users or switchers) of two different groups of OCs: OCs containing DRSP and OCs containing other progestins ("Other OCs" cohort). Each of these two cohorts will be divided in two sub-cohorts: a) the DRSP cohort into a 24-day regimen (DRSP/EE 24d) and a 21-day regimen (DRSP/EE 21d) sub-cohort, and b) the "other progestins" cohort into a sub-cohort of levonorgestrel (LNG)-containing OCs and a sub-cohort of all other progestin-containing OCs. A "non-interference" approach will be used. This means that 1) all patients who are new users of an OC are eligible for enrollment if they give their informed consent and 2) the recruitment of patients should not (significantly) influence the physician's prescribing behavior. This approach is used to provide standardized, comprehensive, reliable information on these groups of OCs under routine medical conditions. In this study, regular, active contacts with the cohort members (= active surveillance) should provide all necessary information on health-related events or changes in health status.

Besides baseline, contacts to obtain information are planned every six month for a maximum of 4 years (48 months after baseline) in Europe and 5 years (54 months after baseline) in the US. By means of these contacts, almost all relevant clinical outcomes will be captured. However, laypersons often misclassify adverse events (e.g., pain in the legs after standing a long period of time as "thrombosis" or migraine attacks as "stroke" even if modern imaging procedures do not provide any indication of the perceived event). This type of inaccuracy in patient reports will require careful validation of the reported events. This will be accomplished by contacting the relevant physicians and by reviewing relevant source documents. Under routine medical conditions, diagnosis of a VTE it is not always confirmed by an imaging procedure. Therefore, reported VTEs have to be classified as "confirmed" or "not confirmed" according to a predefined algorithm. At the end of the study a blinded adjudication will be conducted to verify this classification. Three independent medical experts with experience in VTE will review all available information on the reported VTEs. For this process the brand names, dose, regimen and composition of the OCs will be rendered anonymous. The adjudicators will perform the reviews independently of each other and without knowing the judgment of the other adjudicators or the investigators.

The adjudication procedure will include the following steps:

1) Independent adjudication by the individual specialists

2) Documentation of the individual assessments

3) Comparison of the individual assessments

4) Discussion of "split decisions" among the adjudicators without enforcement of a unanimous decision

5) Independent re-adjudication of the discussed cases by the individual adjudicators

6) Documentation of the individual assessments

The final analysis will be based on a strategy where at least one adjudicator has classified the event as confirmed before the discussion took place, because it represents the most conservative approach. However, alternative analyses can be conducted, if requested by regulatory authorities.

The same procedure will be used for the adjudication of ATE. Based on interim results the independent Safety Monitoring and Advisory Board may decide to use the same adjudication procedure for other outcomes of interest too.

### Study Centers

Recruitment of the cohort members is conducted via a network of approximately 2000 OC prescribing physicians in Europe (Austria, Germany, Poland, and Sweden) and the United States.

The combined cohort will include approx. 80,000 women, with about 50,000 women in the US and 30,000 women in Europe. Study measures should not interfere with the prescribing behavior of physicians or with the individual needs of the participating women. Influence on the preference for specific OCs is to be avoided but significant efforts are to be undertaken to ensure standardized, comprehensive and reliable documentation of all baseline characteristics and adverse events during the follow-up period.

### Study Participants

The study participants are women who

• have a prescription for a new OC

• are willing to participate in this long-term follow-up study

These women can be either OC starters (first-ever user) or OC switchers. There are no specific medical inclusion or exclusion criteria. However, women

• who are not cooperative and/or available for follow-up may be excluded from study participation

• with a language barrier will not be eligible for study inclusion, as all materials are printed in the country-specific language

At the participating centers, all women seeking a prescription for a new OC are to be asked by their physician if they are willing to participate. The physician should explain the nature of the study, its purpose and associated procedures, and the expected duration of follow-up to each woman prior to her study entry. Each woman must have ample opportunity to ask questions and has to be informed about her right to withdraw from the study at any time without disadvantage and without having to provide reasons for her decision. This information will be provided on an informed consent and data privacy form which must be signed by all study participants prior to study entry. These documents are to be approved by the relevant Ethics Committees and the relevant Data Privacy Offices, if applicable.

The whole process of patient information of this study should not start before the discussion and prescription of the new OC has taken place.

Once enrolled, a subject may discontinue the use of her OC at any time. However, subjects will continue to be followed up whether or not they remain on OCs, provided that they do not withdraw their consent. During the follow-up phase, subjects will be asked whether they have discontinued OC use or whether they have switched to another OC preparation. Information on the date and reason for discontinuation or switching during the follow-up phase will also be collected.

### Baseline Survey

Each physician's office will be provided with questionnaires for collecting data at baseline. The baseline visit will take place at the participating physician's office. All women who receive a new prescription for an oral contraceptive are to be asked if they are willing to participate. The physician will not discuss the study with the women until the OC has been prescribed. This ensures that participation in the study is not considered a requirement for treatment. After discussing the study details (including follow-up procedures and intervals, content and duration of follow-up contacts, use of data collected, etc.), each subject will be asked to provide written informed consent to participate in the study. If the subject needs time to consider participation, she can leave the physician's office with her prescription and take an appropriate period of time to decide whether to participate or not. If she decides to take part in this study, she hands in the signed documents at the physicians' office.

Baseline data are to be recorded on a self-administered questionnaire containing questions relating to the participant's state of health, potential risk factors and history of medication and OC use. The patient is also asked to provide her own address, e-mail address and phone number as well as the contact information of a relative or friend for alternative contact in case the study participant can not be reached. In compliance with data protection regulations names, addresses and phone numbers are documented on a separate sheet.

### Follow-up

Patients will be recruited within the first 2 years after the market introduction of DRSP/EE 24d and follow-up is scheduled every 6 month for up to 4 years in Europe and up to 5 years in the United States. The follow-up questionnaires include data on the occurrence of adverse events, exact dates for using, stopping or switching OCs, as well as changes in risk factors relevant to VTE and ATE. Questionnaires will be mailed to the participating women, who often know more about their own personal health related issues than the physician who prescribes their OC. This can especially be true for information on adverse events that are treated by other physicians. Experiences with this study design show, that events may be reported sporadically by the participant or by relatives, friends or attending physicians between the regular follow-ups. These reports will be documented and validated in the same way as regular reports.

A low "lost to follow-up rate" will be essential for the validity of the study. In order to minimize loss to follow-up a multi-faceted, four-level follow-up process will be established. Level1 activities include mailing of the follow-up questionnaire and - in case of no response - two reminder letters. If level1 activities do not lead to a response, multiple attempts are to be made to contact the woman, friends, relatives and the Gynecologist/Primary Care Physician per phone. In parallel to these level2 activities searches in national and international telephone and address directories are started (level3 activities). If this is not successful, an official address search via the respective governmental administration will be conducted. This level4 activity can provide information on new addresses (or emigration or death). If necessary, a search in the national death registers could be started at the end of the study to clarify the vital status of patients who are lost to follow-up after level4 activities. Overall, the loss to follow-up of the combined cohort should be kept at less than 10% of the recruited population.

The follow-up questionnaires will address the occurrence of adverse events. Reasons for switching to another OC or discontinuation will be requested if applicable.

### Validation of Self-Reported Events

Validation of self-reported events begins at the level of the national field work organization with a review of all subjective "events." This is followed by a further review at the international coordinating center (ZEG).

If an event is reported by a participant, the subjectively perceived symptoms, signs, and if possible the diagnoses as understood by the patient should be recorded. The name and address of the relevant physician ought to be provided by the participant.

These report forms are to be immediately passed on to the responsible medical reviewer/group. In case of unclear or missing information, the woman will be contacted by telephone, e-mail or by other means of communication. For many events it may be necessary to contact the diagnosing and/or treating physician for clarification and validation of the information received from the patient. This procedure is mandatory for all serious clinical outcomes (Serious adverse event means any adverse event that results in death, a life-threatening experience, inpatient hospitalization, persistent or significant disability/incapacity, or requires medical/surgical intervention to prevent one of said outcomes). (incl. VTE and ATE).

Under routine medical conditions, diagnosis of a VTE it is not always confirmed by an imaging procedure. Therefore, reported VTE have to be classified as "confirmed" or "not confirmed" according to the following predefined algorithm:

I. Definite VTE: Confirmed by diagnostic measures with high specificity.

DVT: phlebography, duplex sonography, magnetic resonance imaging

PE: pulmonary angiography, ventilation-perfusion scan, spiral computed tomography, magnetic resonance imaging, transesophageal echocardiography

II. Probable VTE: Clinical diagnosis confirmed by a health professional, supported by an unspecific diagnostic test (such as D-dimer for VTE) and/or a subsequent specific therapy (such as fibrinolysis or long-term anticoagulant therapy). However, if the attending physician confirmed the diagnosis, the event will be classified as a probable event even if specific treatment was not given or if positive test results are not available.

III. No VTE:

- VTE excluded by an imaging procedure

- Other medical condition diagnosed by the attending physician

- Woman does not contact a health professional to clarify her symptoms and no diagnostic measures are performed that could clarify the diagnosis

A VTE will be classified as "confirmed" if the diagnosis is categorized as definite or probable according to the above criteria, regardless of hospital admission or type of treatment provided.

At the end of the study this classification will be checked by blind independent adjudication. The Safety Monitoring and Advisory Board will appoint three independent medical experts who will review all available information on the reported VTE. However, brand names, dose, regimen and composition of the OC(s) used by the reporting woman will be rendered anonymous. The adjudicators will perform the review independently of each other and without knowing the judgment of the other adjudicators. If at least one adjudicator classifies a report as confirmed VTE, the reported event will be considered a confirmed VTE (cf. Methods/Design).

### Reporting of Serious and/or unexpected adverse events

ZEG will report all serious and/or unexpected events that are possibly related to the use of any OC to the relevant pharmaceutical companies. A physician on the ZEG study team will assess the likelihood of a causal relationship to OC use for each serious adverse event in accordance with a predefined algorithm.

ZEG will not monitor whether the pharmaceutical companies meet their obligation to report these events to the Health Authorities according to (inter)national rules.

### Data Management

When questionnaires are received from study participants, all pages are counted and date-stamped. Questionnaires are to be checked for correct subject identification number, missing pages, legibility, and incomplete information on the questionnaires. Missing pages, illegible or missing information will be requested from the study participants.

Data are entered by double data entry via formatted entry screens designed to reflect the appearance of the questionnaire. Discrepancies between first and second data entry are identified by comparison of the two entry files within the statistical software SAS. The decision on the true entry is done by the responsible data manager at ZEG. This may require direct contact with the study participant who filled in the questionnaire. Corrections will be made to the questionnaire only after contact with the study participant or her treating physician. All corrections are dated and initialled by the data manager who received the relevant new information (e.g., via direct contact or by a copy of medical reports/documents). The incorrect CRF entry will be crossed out; however, it must remain legible, and the correct entry will be placed next to it. The reason for any correction of medical data on the questionnaire must be documented.

Quality control of entered data will be supported by SAS plausibility programs which include range, coding, missing and date checks as well as cross-reference (consistency) checks between variables.

### Size of the Study

The study was designed to analyze rare events (according to the CIOMS classification 1 - 10 events per 10,000 women years). The adverse events of particular interest are VTE and ATE. Based on the EURAS results the estimated VTE and ATE incidence rates in the young study population are ~9/10,000 women years for VTE and ~2/10,000 WY for ATE.

The primary outcome of interest is the VTE hazard ratio between DRSP/EE 24d and Other OCs. The null hypothesis to be tested is: **HR_VTE _> 2 **(i.e., the VTE hazard ratio for DRSP/EE 24d vs. Other OCs is higher or equal to 2). The alternative hypothesis is: **HR_VTE _< 2**. In a sub-analysis VTE hazard ratios will also be calculated for DRSP/EE 24d vs. LNG-containing OCs.

The 2 to 5 years of follow-up of 80,000 women should result in at least 220,000 documented women-years. Based on the market shares of DRSP-containing and LNG-containing OCs it is expected that the DRSP/EE 24d and LNG exposure will be approx. 44,000 and 22,000 women-years, respectively. Power calculations based on an one-sided alpha of 0.025, a statistical power of 90%, and the VTE incidence given above showed that approximately 90,000 women-years would be needed to show non-inferiority of DRSP/EE 24d versus "Other OCs". In addition, approx. 42,600 and 21,300 women-years of DRSP/EE 24d and LNG exposure would be needed to show non-inferiority of DRSP/EE 24d versus LNG-containing OCs (i.e., slightly less than the expected exposure of 44,000 and 22,000 women-years). Furthermore, the study will have a statistical power of 99% and 75% to exclude a threefold and twofold risk of ATE, respectively.

In essence, the study is powered to exclude a twofold risk of VTE and a threefold risk of ATE - if the true incidence for the outcomes of interest is not different for the two cohorts. The EURAS study, however, showed a low ATE incidence for DRSP-containing OCs. If the INAS OC study shows a similar low ATE incidence for DRSP/EE 24d the power of the study will be sufficient to exclude a twofold ATE risk for DRSP/EE 24d.

These power calculations suggest that the INAS OC study is sufficiently powered to show non-inferiority of DRSP/EE 24d compared to established OCs (including LNG-containing OCs). However, exact power calculations based on actual incidences and drop-out rates should be done on the basis of one year of follow-up data. If these calculations do not confirm the assumed incidences and drop-out rates the independent Safety Monitoring and Advisory Board (SMAB) may discuss the need for adapting patient numbers and follow-up times.

### Data Analysis

It is feasible to analyze the data for the US and the European part separately and draw conclusions from it.

The final analyses will include both an "as treated" (AT) and where necessary also an intention-to-treat (ITT) analysis. The safety conclusions of the study, however, will be based on the AT analyses because the ITT approach potentially dilutes differences between treatments.

Crude as well as adjusted hazard ratios will be calculated. The appropriate confounding variables will be built into the model. Based on the expectation of a small absolute number of serious outcomes of interest the number of confounding variables will be limited to well established risk factors for these outcomes (e.g., age, BMI, duration of use, and VTE history). The final decision on the confounding variables will be made by the Safety Monitoring and Advisory Board before the first interim analysis of follow-up data. In addition, alternative analysis will be performed with other potential baseline risks to check the appropriateness of this decision.

The principal investigator will present a detailed analysis plan to the independent Safety Monitoring and Advisory Board for approval before the first interim analysis of follow-up data.

### Safety Monitoring and Advisory Council

This study will maintain scientific independence and will be governed by an independent Safety Monitoring and Advisory Board throughout the study time. The council is concerned with the safety of the oral contraceptives used in this study and the protection of the public. Commercial interests must not supersede the ethical principles of nonmaleficence (obligation not to inflict harm intentionally) and beneficence (obligation to contribute to the welfare of the patient). SMAC has full authority over the study. This includes approval of study protocol, final study report and publications emerging from the study, as well as stopping the study for safety reasons, if necessary.

Bayer Schering Pharma AG provided an unconditional grant. The Berlin Center for Epidemiology and Health Research (ZEG), Germany and its investigator team will be accountable to the Safety Monitoring and Advisory Board in all scientific matters. The investigators update the SMAC members on study status and interim results at least twice a year. SMAC's conclusions and decisions are made in executive session without attendance of the investigators or representatives of Bayer Schering Pharma AG.

The SMAC members (Samuel Shapiro (South Africa, Chair), David Grimes (United States), Edward Pritchett (United States), Andrea Rapkin (United States), Anne Szarewski (UK), Carolyn Westhoff (United States)) are international experts in relevant scientific fields (e.g., epidemiology, gynecology and cardiology). Specific questions (legal, ethical, regulatory, etc.) may be addressed by ad-hoc consultants. The members of the council will receive remuneration of expenses and an honorarium to compensate for loss of potential earnings during their work for SMAC. The members will not be involved in or paid for the operational conduct of the study.

### Study Management

This study will be conducted in accordance with

➢ 'Guidelines for Good Pharmacoepidemiology Practices (GPP)' issued by the International Society for Pharmacoepidemiology in 2004

➢ 'Good Epidemiological Practice (GEP) - Proper Conduct in Epidemiologic Research' issued by the European Epidemiology Federation in 2004

➢ The ethical principles that have their origin in the Declaration of Helsinki.

All processes that are relevant for legal compliance of the study or the integrity of the data are subject to quality control measures. This includes the development of study protocols, questionnaires, databases and data entry screens, the data entry, plausibility checks, validation of clinical outcomes, reporting of adverse drug reactions, data analysis, report writing, publications of results, archiving. The quality control measures are based on the four-eye principle (i.e., each work process that is relevant for the overall quality of the study has to be quality controlled by independent second person).

As an additional quality control, the independent Safety Monitoring and Advisor Board will supervise the study.

### Ethics and Privacy

The study starts after all relevant legal, administrative and ethical requirements have been fulfilled. Information on the identity of the patients and treating physicians will be kept separate from the clinical information throughout the study. Confidentiality of information on study subjects will be maintained. All relevant national data protection laws will be followed. The study protocol will be submitted to the relevant Ethics Committees, Institutional Review Boards and regulatory authorities for comments and approval.

The study subjects will not be placed at risk as a result of the study. OC prescription takes place independently of study participation.

Participating physicians will not receive payments for their time and labor but only for the running costs of their practice (salaries of staff, maintenance of equipment, cleaning, rent, etc.). All payments will be completely documented and will be only based on work actually performed.

### Publications

The final study protocol and the results of this study will be published. In accordance with the International Committee of Medical Journal Editors (ICMJE) initiative requiring prior entry of clinical studies in a public registry as a condition for publication, the study will be registered in the U.S. National Institutes of Health's protocol registration database http://ClinicalTrials.gov.

The manuscripts will be approved by the Safety Monitoring and Advisory Board before submission. Bayer Schering Pharma AG has no right to prevent the publication of results or to influence the interpretation of data.

## Discussion

The feasibility of the study is considered to be very high because of its similar design to the EURAS-OC study. All relevant methodological and logistical features of the study were successfully tested in the EURAS study. E.g., in the EURAS-OC study a very low loss to follow-up rate of 2.4% was achieved (cf. Follow-up). Therefore, a loss to follow-up rate in this study of less than 10% is expected.

This is a non-interventional, prospective cohort study with the limitations inherent to non-experimental research. I.e., the possibility of bias and residual confounding can never be entirely eliminated, and the ability to infer causation is correspondingly limited [20]. Today, improved insight into potential sources of bias and confounding as well as refinements of statistical and epidemiologic methodology helps to estimate the impact of bias and residual confounding. However, the difficulty may remain unresolved when all that exists is a weak association. In practical terms, a point in the gradient of declining relative risk must be reached at which the amount of bias and residual confounding becomes so small that it cannot realistically be ruled out [21]. Hazard ratio estimates that are close to unity may not allow differentiation between causation, bias and confounding.

Different epidemiological methods vary in their susceptibility to different kinds of bias. In the context of this study - a prospective, controlled, non-interventional cohort study - the focus here is on the role of detection bias. It is conceivable that the safety information given to physicians and patients influences the frequency and accuracy of diagnostic measures for VTE. It is also conceivable that patients using a newly introduced product are more carefully monitored. If so, this may lead to the detection of otherwise occult VTE cases.

It should be noted here that residual confounding in this study - as in every other non-interventional study - cannot be completely excluded. Although all confounders known for the individual women are documented in detail at baseline adjustment or stratification cannot be done for unknown confounders.

However, the chosen design minimizes the impact of referral and misclassification bias, healthy user effect and loss to follow-up. Overall, it is expected that the study design is robust enough to interpret hazard ratios of 1.5 or higher.

## Abbreviations

AMI: Acute Myocardial Infarction; AT: As Treated; ATE: Arterial Thromboembolism; BMI: Body Mass Index; CRF: Case Report Form; CVA: Cerebrovascular Accident; DRSP: Drospirenone; DVT: Deep Venous Thrombosis; EE: Ethinylestradiol; EURAS: European Active Surveillance (study); GEP: Good Epidemiological Practice; GPP: Guideline for Good Pharmacoepidemiology Practices; HR: Hazard Ratio; ICMJE: International Committee of Medical Journal Editors; INAS: International Active Surveillance; ITT: Intention To Treat; LNG: Levonorgestrel; OC: Oral Contraceptive; PE: Pulmonary Embolism; SMAB: Safety Monitoring and Advisory Board; VTE: Venous Thromboembolism; WY: Women-years; ZEG: Berlin Center for Epidemiology & Health Research (acronym for the German term 'Zentrum für Epidemiologie & Gesundheitsforschung')

## Competing interests

The authors declare that they have no competing interests.

## Authors' contributions

JCD and AA developed the design of the study, KB drafted the manuscript. All authors read and approved the final manuscript.

## Pre-publication history

The pre-publication history for this paper can be accessed here:

http://www.biomedcentral.com/1471-2288/9/77/prepub

## References

[B1] World Health Organization Collaborative Study of Cardiovascular Disease and Steroid Hormone Contraception Effect of different progestagens in low oestrogen oral contraceptives on venous thromboembolic disease Lancet 1995 346 1582 8 7500749

[B2] Jick H Jick SS Gurewich V Myers MW Vasilakis C Risk of idiopathic cardiovascular death and nonfatal venous thromboembolism in women using oral contraceptives with differing progestagen components Lancet 1995 346 1589 93 10.1016/S0140-6736(95)91928-7 7500750

[B3] Spitzer WO Lewis MA Heinemann LA Thorogood M MacRae KD Third generation oral contraceptives and risk of venous thromboembolic disorders: an international case-control study. Transnational Research Group on Oral Contraceptives and the Health of Young Women BMJ 1996 312 83 8 8555935 10.1136/bmj.312.7023.83 PMC2349742

[B4] Bloemenkamp KW Rosendaal FR Helmerhorst FM Buller HR Vandenbroucke JP Enhancement by factor V Leiden mutation of risk of deep-vein thrombosis associated with oral contraceptives containing a third-generation progestagen Lancet 1995 346 1593 6 10.1016/S0140-6736(95)91929-5 7500751

[B5] Kemmeren JM Algra A Grobbee DE Third generation oral contraceptives and risk of venous thrombosis: meta-analysis BMJ 2001 323 131 4 10.1136/bmj.323.7305.131 11463678 PMC34722

[B6] Heinemann LA Lewis MA Assmann A Thiel C Case control studies on venous thromboembolism: bias due to design? A methodological study on venous thromboembolism and steroid hormone use Contraception 2002 65 207 14 10.1016/S0010-7824(01)00309-2 11929642

[B7] Heinemann LA The changing scene: an unnecessary pill crisis Hum Reprod Update 1999 5 746 55 10.1093/humupd/5.6.746 10652983

[B8] Lewis MA MacRae KD Kuhl-Habich D The differential risk of oral contraceptives: the impact of full exposure history Hum Repod 1999 14 1493 9 10.1093/humrep/14.6.1493 10359554

[B9] Vandenbroucke JP Helmerhorst FM Bloemenkamp KW Third generation oral contraceptive and deep venous thrombosis: from epidemiologic controversy to new insight in coagulation Am J Obstet Gynecol 1997 177 887 91 10.1016/S0002-9378(97)70289-8 9369840

[B10] Crosignani PG La Vecchia C Concordant and discordant effects on cardiovascular risks exerted by oestrogen and progestogens in women using oral contraception and hormone replacement therapy: ESHRE Capri Workshop Group Hum Reprod Update 1999 5 681 7 10.1093/humupd/5.6.681 10652978

[B11] Spitzer WO Faith JM MacRae KD Myocardial infarction and third generation oral contraceptives: aggregation of recent studies Hum Reprod 2002 17 9 2307 14 10.1093/humrep/17.9.2307 12202417

[B12] Dinger J Heinemann AJ Kuehl-Habich D The safety of a drospirenone-containing oral contraceptive: final results from the European Active Surveillance study on Oral Contraceptives based on 142,475 women-years of observation Contraception 2007 75 344 354 10.1016/j.contraception.2006.12.019 17434015

[B13] Seeger JD Loughlin J Eng PM Clifford CR Cutone J Walker AM Risk of Thromboembolism in Women Taking Ethinylestradiol/Drospirenone and Other Oral Contraceptives Obstetrics & Gynecology 2007 110 587 593 10.1097/01.AOG.0000279448.62221.a8 17766604

[B14] Van Hylckama Vlieg A Helmerhorst FM Vandenbroucke JP Doggen CJM Rosendaal FR The venous thrombotic risk of oral contraceptives, effects of oestrogen dose and progestogen type: results of the MEGA case-control study BMJ 2009 339 b2921 10.1136/bmj.b2921 19679614 PMC2726929

[B15] Lidegaard Ø Løkkegaard E Svendsen AL Agger C Hormonal contraception and risk of venous thromboembolism: national follow-up study BMJ 2009 339 b2890 10.1136/bmj.b2890 19679613 PMC2726928

[B16] Dinger JC Voigt K Moehner S Case-control study: use of oral contraceptives containing dienogest and risk of venous thromboembolism Pharmacoepidemiol Drug Saf 2009 18 S114

[B17] Dinger J Oral contraceptives and venous thromboembolism: old questions revisited J Fam Plann Reprod Health Care 2009 35 211 213 10.1783/147118909789587385 19849911

[B18] Klipping C Duijkers I Trummer D Marr J Suppression of ovarian activity with a drospirenone-containing oral contraceptive in a 24/4 regimen Contraception 2008 78 16 25 10.1016/j.contraception.2008.02.019 18555813

[B19] Krattenmacher R Drospirenone: pharmacology and pharmacokinetics of a unique progestogen Contraception 2000 62 29 38 10.1016/S0010-7824(00)00133-5 11024226

[B20] Susser M What is a cause and how do we know one? A grammar for pragmatic epidemiology Am J Epidemiol 1991 133 635 48 2018019 10.1093/oxfordjournals.aje.a115939

[B21] Shapiro S Bias in the evaluation of low-magnitude associations: an empirical perspective Am J Epidemiol 2000 151 939 945 10853631 10.1093/oxfordjournals.aje.a010135

